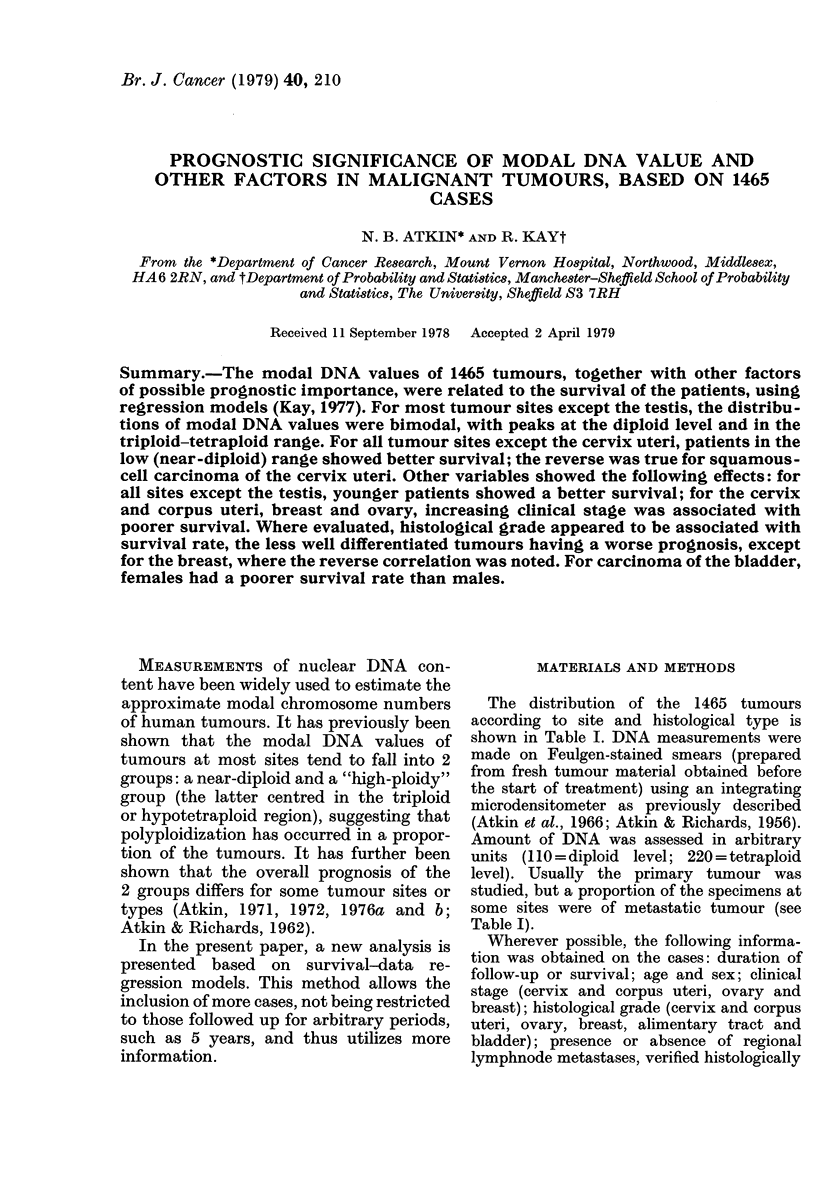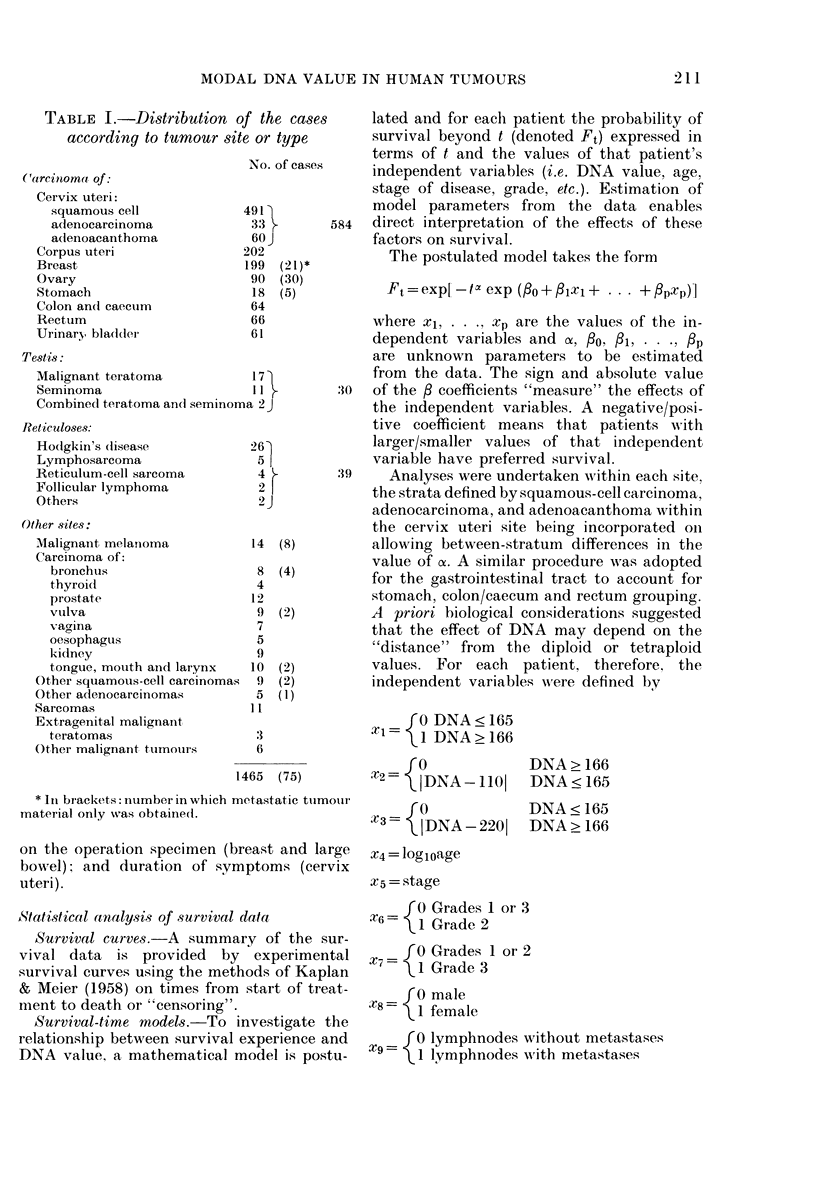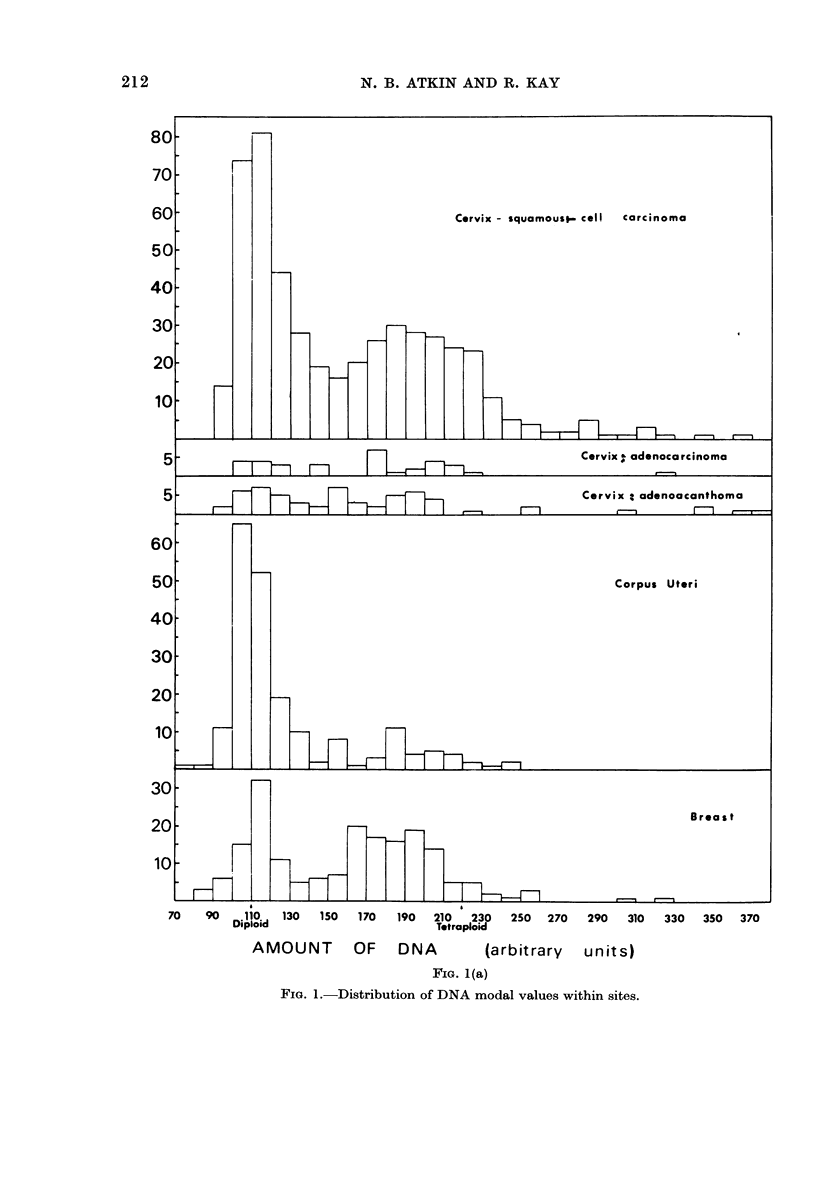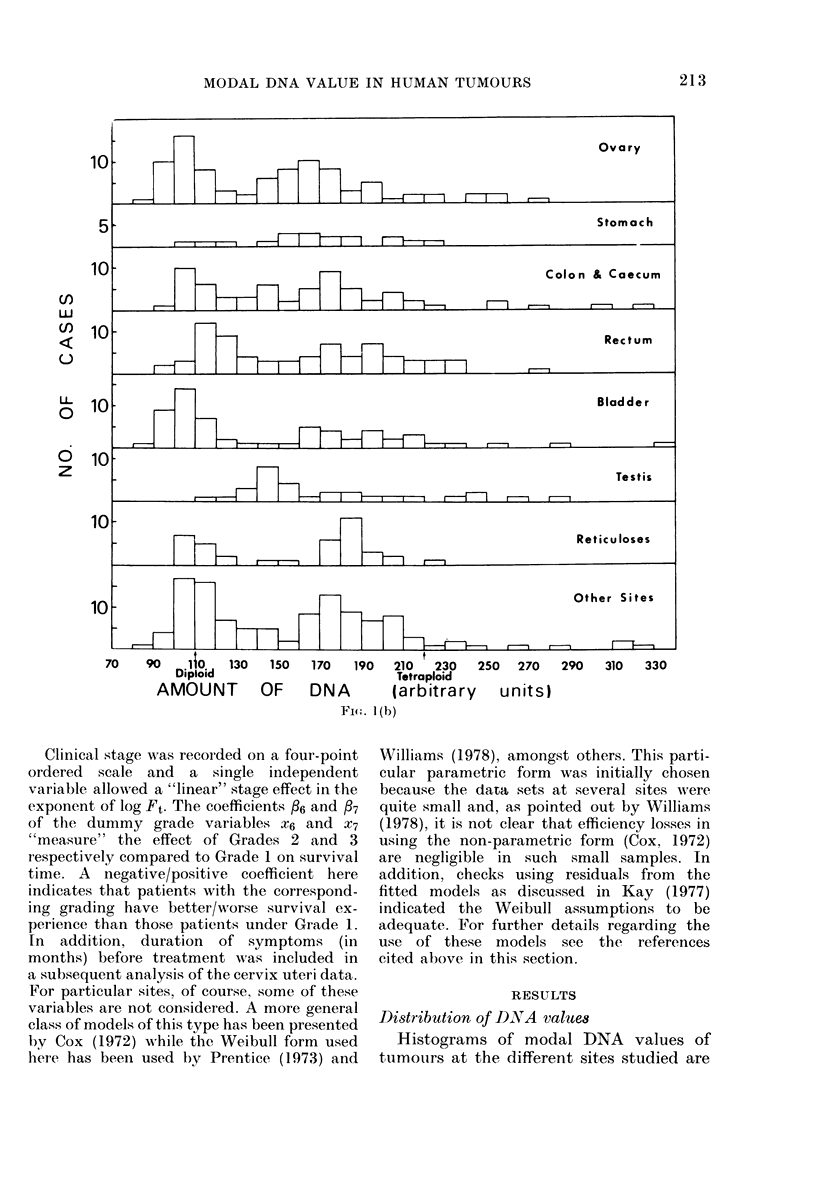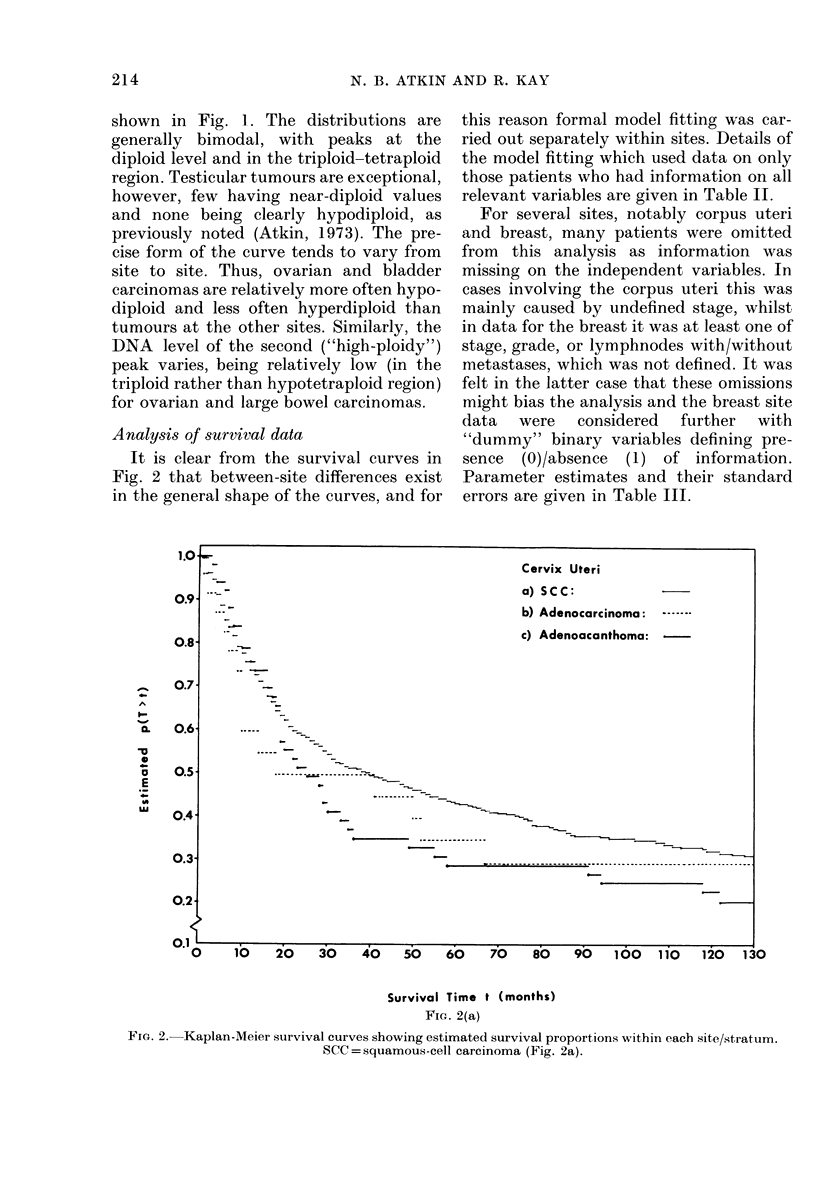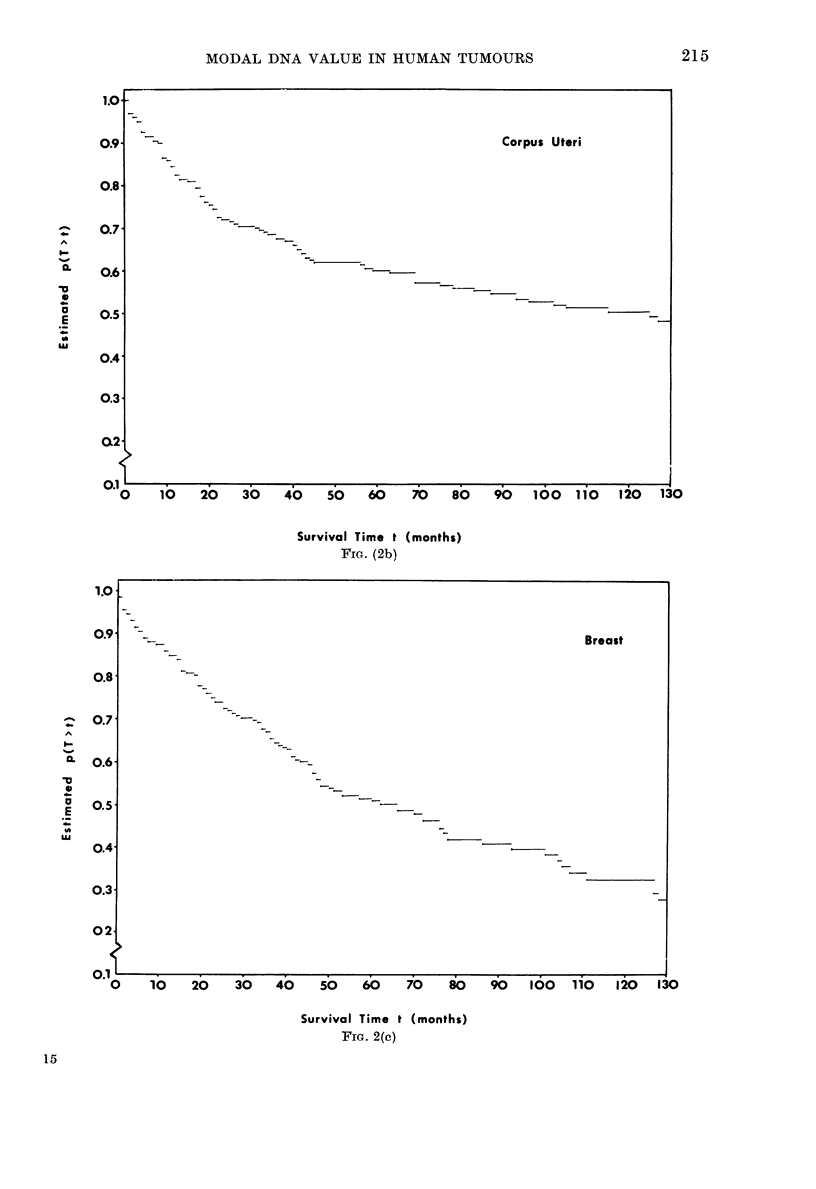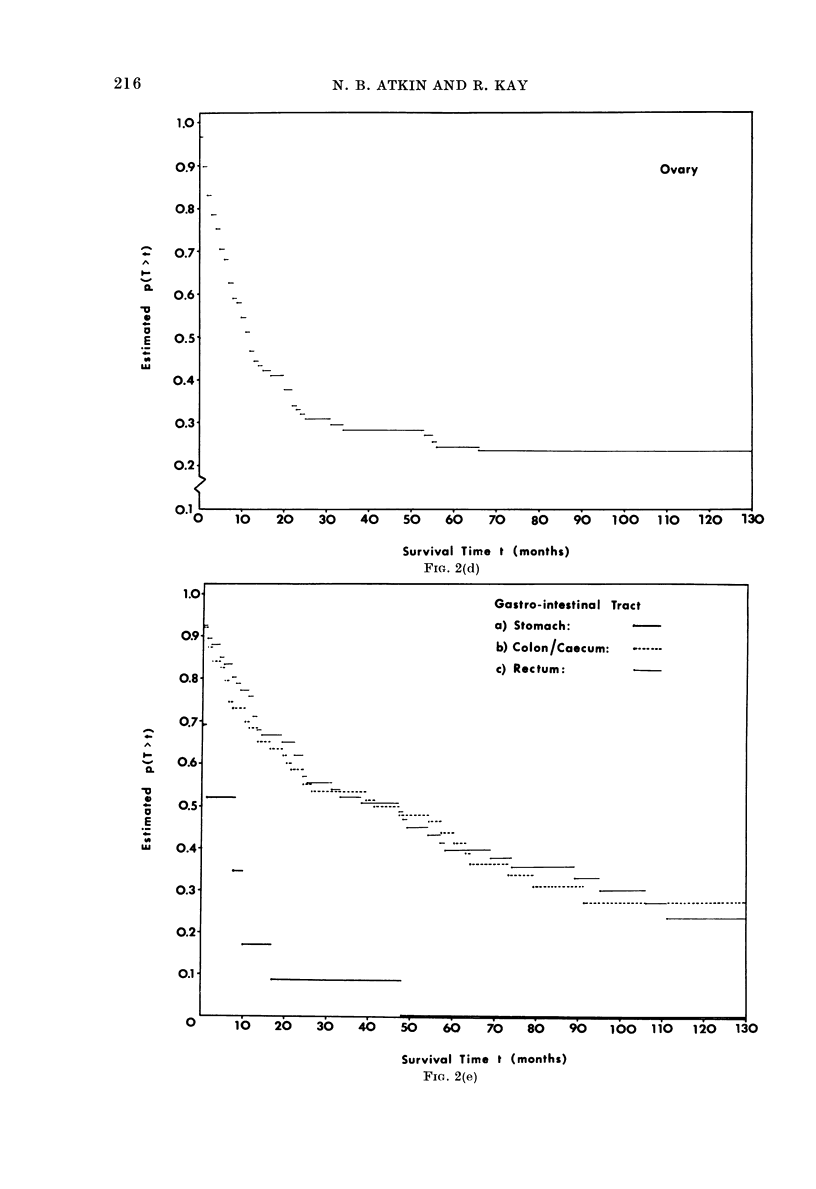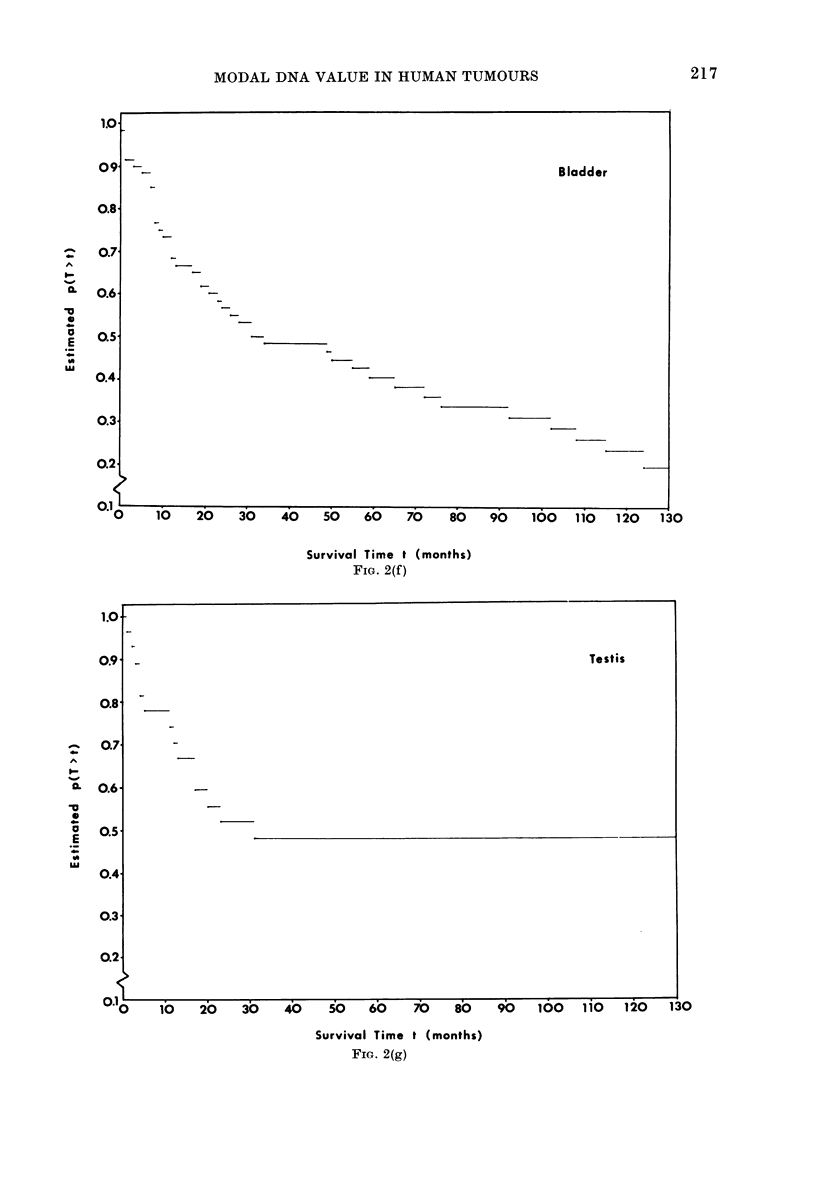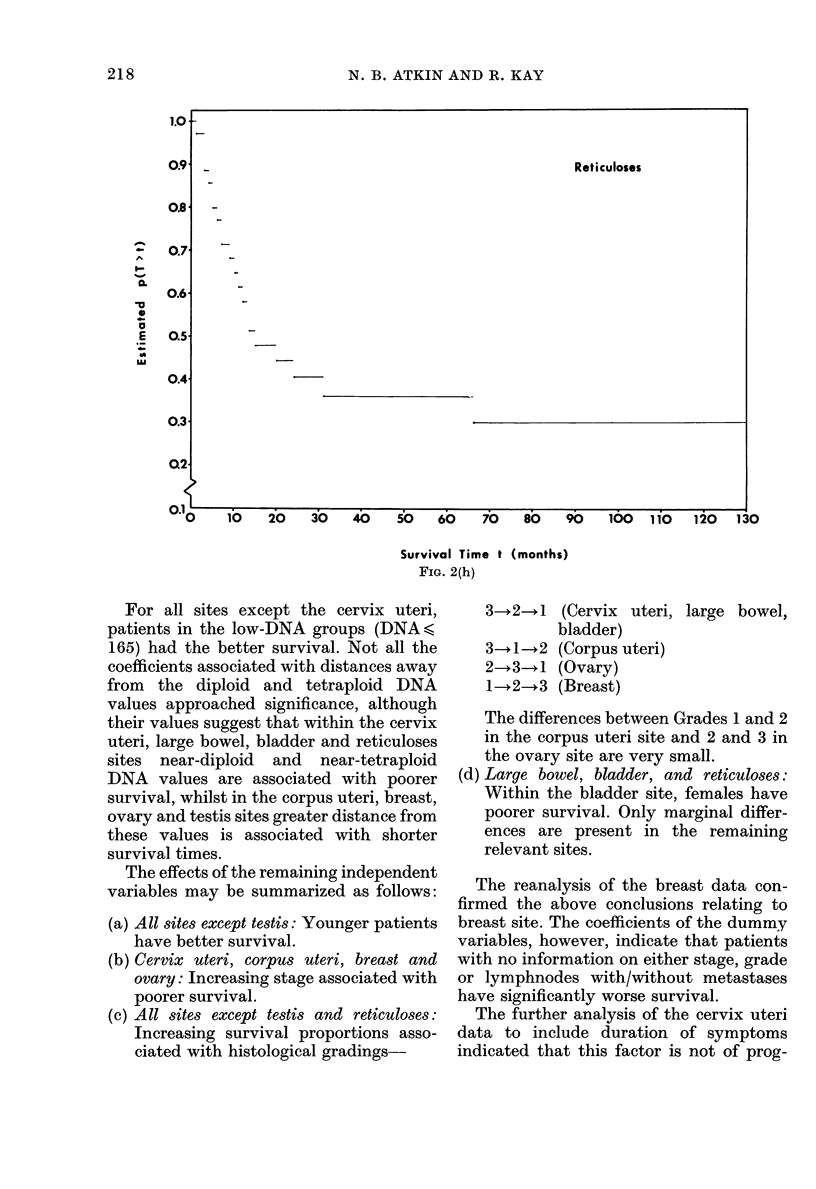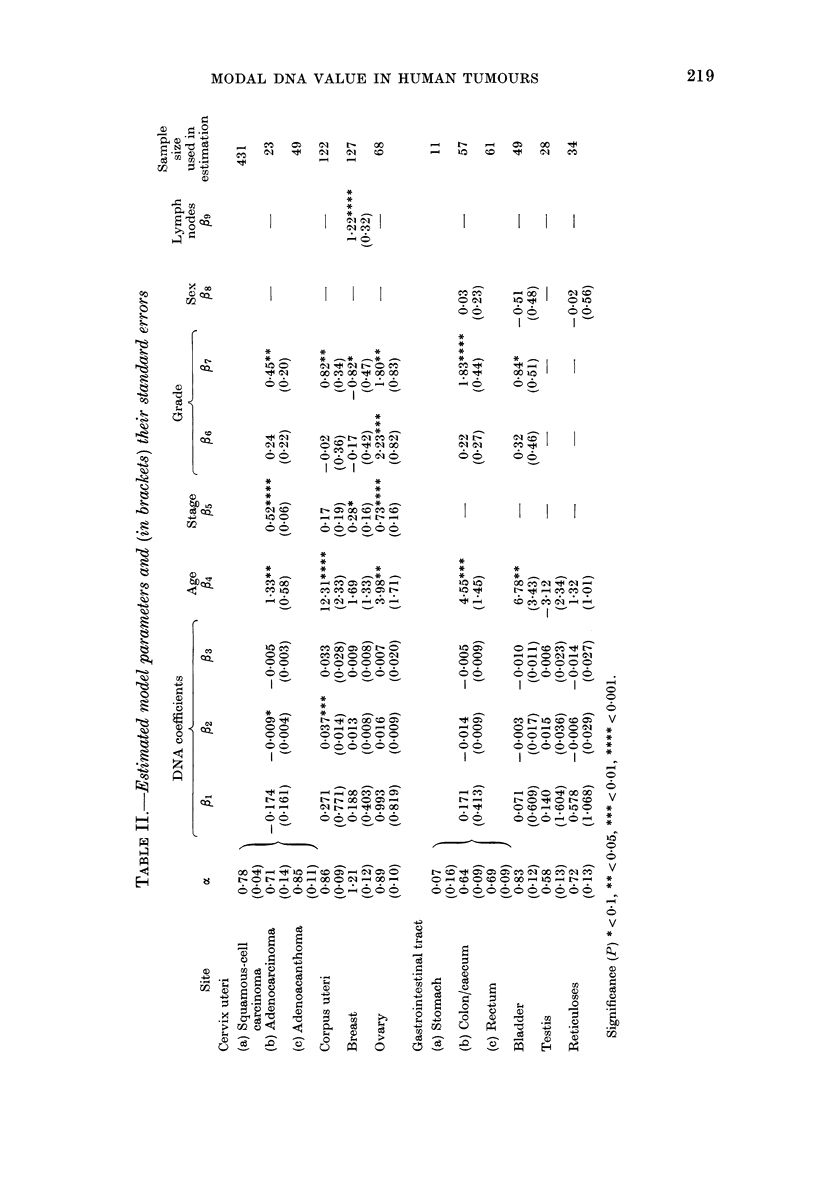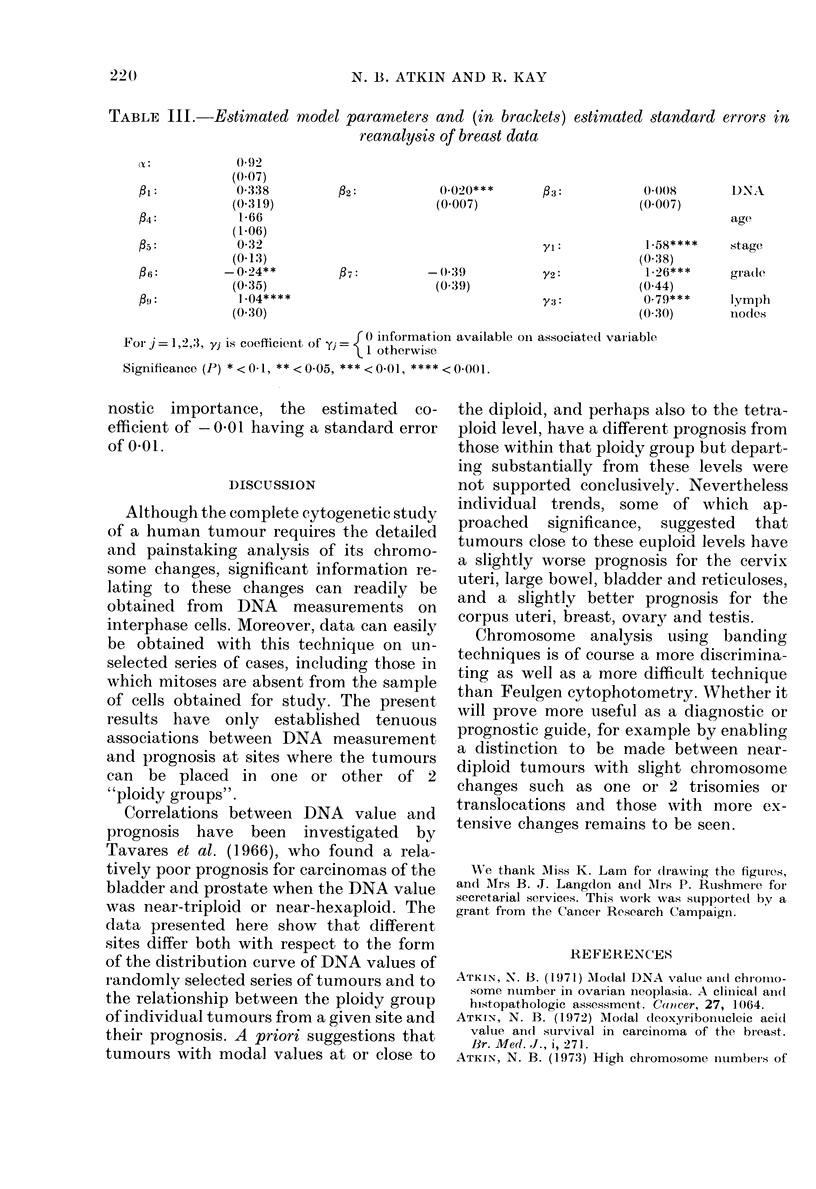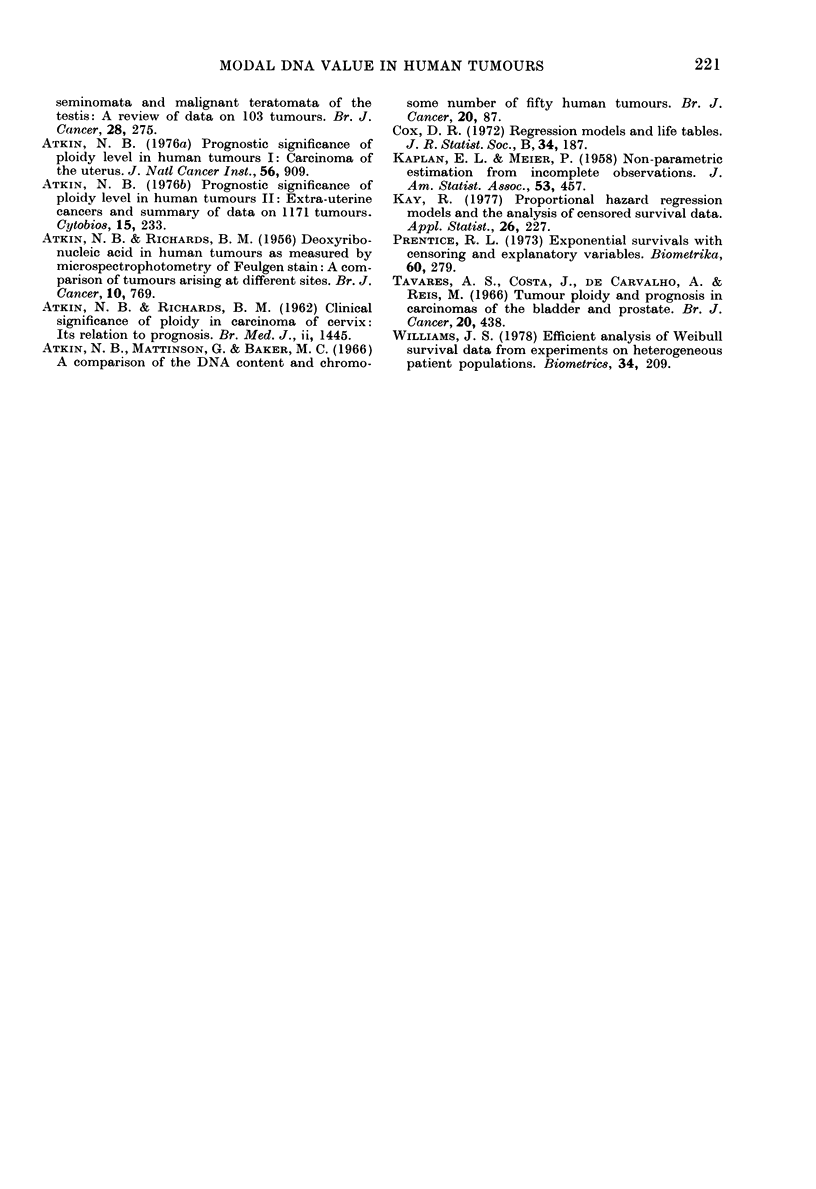# Prognostic significance of modal DNA value and other factors in malignant tumours, based on 1465 cases.

**DOI:** 10.1038/bjc.1979.168

**Published:** 1979-08

**Authors:** N. B. Atkin, R. Kay

## Abstract

The modal DNA values of 1465 tumours, together with other factors of possible prognostic importance, were related to the survival of the patients, using regression models (Kay, 1977). For most tumour sites except the testis, the distributions of modal DNA values were bimodal, with peaks at the diploid level and in the triploid-tetraploid range. For all tumour sites except the cervix uteri, patients in the low (near-diploid) range showed better survival; the reverse was true for squamous-cell carcinoma of the cervix uteri. Other variables showed the following effects: for all sites except the testis, younger patients showed a better survival; for the cervix and corpus uteri, breast and ovary, increasing clinical stage was associated with poorer survival. Where evaluated, histological grade appeared to be associated with survival rate, the less well differentiated tumours having a worse prognosis, except for the breast, where the reverse correlation was noted. For carcinoma of the bladder, females and a poorer survival rate than males.


*Br. J. Cancer* (1979) **40**, 210

### PROGNOSTIC SIGNIFICANCE OF MODAL DNA VALUE AND OTHER FACTORS IN MALIGNANT TUMOURS, BASED ON 1465 CASES

N. B. ATKIN* AND R. KAY†

*From the *Department of Cancer Research, Mount Vernon Hospital, Northwood, Middlesex, HA6 2RN, and †Department of Probability and Statistics, Manchester–Sheffield School of Probability and Statistics, The University, Sheffield S3 7RH*

Received 11 September 1978 Accepted 2 April 1979

**Summary.—The modal DNA values of 1465 tumours, together with other factors of possible prognostic importance, were related to the survival of the patients, using regression models (Kay, 1977). For most tumour sites except the testis, the distributions of modal DNA values were bimodal, with peaks at the diploid level and in the triploid–tetraploid range. For all tumour sites except the cervix uteri, patients in the low (near-diploid) range showed better survival; the reverse was true for squamous-cell carcinoma of the cervix uteri. Other variables showed the following effects: for all sites except the testis, younger patients showed a better survival; for the cervix and corpus uteri, breast and ovary, increasing clinical stage was associated with poorer survival. Where evaluated, histological grade appeared to be associated with survival rate, the less well differentiated tumours having a worse prognosis, except for the breast, where the reverse correlation was noted. For carcinoma of the bladder, females had a poorer survival rate than males.**

MEASUREMENTS of nuclear DNA content have been widely used to estimate the approximate modal chromosome numbers of human tumours. It has previously been shown that the modal DNA values of tumours at most sites tend to fall into 2 groups: a near-diploid and a "high-ploidy" group (the latter centred in the triploid or hypotetraploid region), suggesting that polyploidization has occurred in a proportion of the tumours. It has further been shown that the overall prognosis of the 2 groups differs for some tumour sites or types (Atkin, 1971, 1972, 1976*a* and *b*; Atkin & Richards, 1962).

In the present paper, a new analysis is presented based on survival–data regression models. This method allows the inclusion of more cases, not being restricted to those followed up for arbitrary periods, such as 5 years, and thus utilizes more information.

### MATERIALS AND METHODS

The distribution of the 1465 tumours according to site and histological type is shown in Table I. DNA measurements were made on Feulgen-stained smears (prepared from fresh tumour material obtained before the start of treatment) using an integrating microdensitometer as previously described (Atkin *et al.*, 1966; Atkin & Richards, 1956). Amount of DNA was assessed in arbitrary units (110 = diploid level; 220 = tetraploid level). Usually the primary tumour was studied, but a proportion of the specimens at some sites were of metastatic tumour (see Table I).

Wherever possible, the following information was obtained on the cases: duration of follow-up or survival; age and sex; clinical stage (cervix and corpus uteri, ovary and breast); histological grade (cervix and corpus uteri, ovary, breast, alimentary tract and bladder); presence or absence of regional lymphnode metastases, verified histologically

TABLE I.—*Distribution of the cases according to tumour site or type*

| | No. of cases | | |
|---|---|---|---|
| *Carcinoma of:* | | | |
| Cervix uteri: | | | |
| squamous cell | 491 | | 584 |
| adenocarcinoma | 33 | | |
| adenoacanthoma | 60 | | |
| Corpus uteri | 202 | | |
| Breast | 199 | (21)* | |
| Ovary | 90 | (30) | |
| Stomach | 18 | (5) | |
| Colon and caecum | 64 | | |
| Rectum | 66 | | |
| Urinary bladder | 61 | | |
| *Testis:* | | | |
| Malignant teratoma | 17 | | 30 |
| Seminoma | 11 | | |
| Combined teratoma and seminoma | 2 | | |
| *Reticuloses:* | | | |
| Hodgkin's disease | 26 | | 39 |
| Lymphosarcoma | 5 | | |
| Reticulum-cell sarcoma | 4 | | |
| Follicular lymphoma | 2 | | |
| Others | 2 | | |
| *Other sites:* | | | |
| Malignant melanoma | 14 | (8) | |
| Carcinoma of: | | | |
| bronchus | 8 | (4) | |
| thyroid | 4 | | |
| prostate | 12 | | |
| vulva | 9 | (2) | |
| vagina | 7 | | |
| oesophagus | 5 | | |
| kidney | 9 | | |
| tongue, mouth and larynx | 10 | (2) | |
| Other squamous-cell carcinomas | 9 | (2) | |
| Other adenocarcinomas | 5 | (1) | |
| Sarcomas | 11 | | |
| Extragenital malignant teratomas | 3 | | |
| Other malignant tumours | 6 | | |
| | 1465 | (75) | |

* In brackets: number in which metastatic tumour material only was obtained.

on the operation specimen (breast and large bowel); and duration of symptoms (cervix uteri).

*Statistical analysis of survival data*

*Survival curves.*—A summary of the survival data is provided by experimental survival curves using the methods of Kaplan & Meier (1958) on times from start of treatment to death or "censoring".

*Survival-time models.*—To investigate the relationship between survival experience and DNA value, a mathematical model is postulated and for each patient the probability of survival beyond $t$ (denoted $F_t$) expressed in terms of $t$ and the values of that patient's independent variables (*i.e.* DNA value, age, stage of disease, grade, *etc.*). Estimation of model parameters from the data enables direct interpretation of the effects of these factors on survival.

The postulated model takes the form

$$F_t = \exp[-t^{\alpha} \exp(\beta_0 + \beta_1 x_1 + \ldots + \beta_p x_p)]$$

where $x_1, \ldots, x_p$ are the values of the independent variables and $\alpha, \beta_0, \beta_1, \ldots, \beta_p$ are unknown parameters to be estimated from the data. The sign and absolute value of the $\beta$ coefficients "measure" the effects of the independent variables. A negative/positive coefficient means that patients with larger/smaller values of that independent variable have preferred survival.

Analyses were undertaken within each site, the strata defined by squamous-cell carcinoma, adenocarcinoma, and adenoacanthoma within the cervix uteri site being incorporated on allowing between-stratum differences in the value of $\alpha$. A similar procedure was adopted for the gastrointestinal tract to account for stomach, colon/caecum and rectum grouping. *A priori* biological considerations suggested that the effect of DNA may depend on the "distance" from the diploid or tetraploid values. For each patient, therefore, the independent variables were defined by

$$x_1 = \begin{cases} 0 & \text{DNA} \leq 165 \\ 1 & \text{DNA} \geq 166 \end{cases}$$

$$x_2 = \begin{cases} 0 & \text{DNA} \geq 166 \\ |\text{DNA} - 110| & \text{DNA} \leq 165 \end{cases}$$

$$x_3 = \begin{cases} 0 & \text{DNA} \leq 165 \\ |\text{DNA} - 220| & \text{DNA} \geq 166 \end{cases}$$

$$x_4 = \log_{10}\text{age}$$

$$x_5 = \text{stage}$$

$$x_6 = \begin{cases} 0 & \text{Grades 1 or 3} \\ 1 & \text{Grade 2} \end{cases}$$

$$x_7 = \begin{cases} 0 & \text{Grades 1 or 2} \\ 1 & \text{Grade 3} \end{cases}$$

$$x_8 = \begin{cases} 0 & \text{male} \\ 1 & \text{female} \end{cases}$$

$$x_9 = \begin{cases} 0 & \text{lymphnodes without metastases} \\ 1 & \text{lymphnodes with metastases} \end{cases}$$

Fig. 1(a)

Fig. 1.—Distribution of DNA modal values within sites.

FIG. 1(b)

Clinical stage was recorded on a four-point ordered scale and a single independent variable allowed a "linear" stage effect in the exponent of log $F_t$. The coefficients $\beta_6$ and $\beta_7$ of the dummy grade variables $x_6$ and $x_7$ "measure" the effect of Grades 2 and 3 respectively compared to Grade 1 on survival time. A negative/positive coefficient here indicates that patients with the corresponding grading have better/worse survival experience than those patients under Grade 1. In addition, duration of symptoms (in months) before treatment was included in a subsequent analysis of the cervix uteri data. For particular sites, of course, some of these variables are not considered. A more general class of models of this type has been presented by Cox (1972) while the Weibull form used here has been used by Prentice (1973) and Williams (1978), amongst others. This particular parametric form was initially chosen because the data sets at several sites were quite small and, as pointed out by Williams (1978), it is not clear that efficiency losses in using the non-parametric form (Cox, 1972) are negligible in such small samples. In addition, checks using residuals from the fitted models as discussed in Kay (1977) indicated the Weibull assumptions to be adequate. For further details regarding the use of these models see the references cited above in this section.

### RESULTS

### *Distribution of DNA values*

Histograms of modal DNA values of tumours at the different sites studied are shown in Fig. 1. The distributions are generally bimodal, with peaks at the diploid level and in the triploid–tetraploid region. Testicular tumours are exceptional, however, few having near-diploid values and none being clearly hypodiploid, as previously noted (Atkin, 1973). The precise form of the curve tends to vary from site to site. Thus, ovarian and bladder carcinomas are relatively more often hypodiploid and less often hyperdiploid than tumours at the other sites. Similarly, the DNA level of the second ("high-ploidy") peak varies, being relatively low (in the triploid rather than hypotetraploid region) for ovarian and large bowel carcinomas.

*Analysis of survival data*

It is clear from the survival curves in Fig. 2 that between-site differences exist in the general shape of the curves, and for this reason formal model fitting was carried out separately within sites. Details of the model fitting which used data on only those patients who had information on all relevant variables are given in Table II.

For several sites, notably corpus uteri and breast, many patients were omitted from this analysis as information was missing on the independent variables. In cases involving the corpus uteri this was mainly caused by undefined stage, whilst in data for the breast it was at least one of stage, grade, or lymphnodes with/without metastases, which was not defined. It was felt in the latter case that these omissions might bias the analysis and the breast site data were considered further with "dummy" binary variables defining presence (0)/absence (1) of information. Parameter estimates and their standard errors are given in Table III.

FIG. 2(a)

FIG. 2.—Kaplan-Meier survival curves showing estimated survival proportions within each site/stratum. SCC = squamous-cell carcinoma (Fig. 2a).

Corpus Uteri

Estimated p(T > t)

1.0

0.9

0.8

0.7

0.6

0.5

0.4

0.3

0.2

0.1

0 10 20 30 40 50 60 70 80 90 100 110 120 130

Survival Time t (months)

FIG. (2b)

Breast

Estimated p(T > t)

1.0

0.9

0.8

0.7

0.6

0.5

0.4

0.3

0.2

0.1

0 10 20 30 40 50 60 70 80 90 100 110 120 130

Survival Time t (months)

FIG. 2(c)

Ovary

Estimated p(T > t)

1.0

0.9

0.8

0.7

0.6

0.5

0.4

0.3

0.2

0.1

0

10

20

30

40

50

60

70

80

90

100

110

120

130

Survival Time t (months)

FIG. 2(d)

Gastro-intestinal Tract
a) Stomach:
b) Colon/Caecum:
c) Rectum:
Estimated p(T > t)
1.0
0.9
0.8
0.7
0.6
0.5
0.4
0.3
0.2
0.1
0
10
20
30
40
50
60
70
80
90
100
110
120
130
Survival Time t (months)

FIG. 2(e)

Bladder

Estimated p(T > t)

1.0

0.9

0.8

0.7

0.6

0.5

0.4

0.3

0.2

0.1

0

10

20

30

40

50

60

70

80

90

100

110

120

130

Survival Time t (months)

FIG. 2(f)

Testis

Estimated p(T > t)

1.0

0.9

0.8

0.7

0.6

0.5

0.4

0.3

0.2

0.1

0

10

20

30

40

50

60

70

80

90

100

110

120

130

Survival Time t (months)

FIG. 2(g)

FIG. 2(h)

For all sites except the cervix uteri, patients in the low-DNA groups (DNA ⩽ 165) had the better survival. Not all the coefficients associated with distances away from the diploid and tetraploid DNA values approached significance, although their values suggest that within the cervix uteri, large bowel, bladder and reticuloses sites near-diploid and near-tetraploid DNA values are associated with poorer survival, whilst in the corpus uteri, breast, ovary and testis sites greater distance from these values is associated with shorter survival times.

The effects of the remaining independent variables may be summarized as follows:

(a) *All sites except testis:* Younger patients have better survival.

(b) *Cervix uteri, corpus uteri, breast and ovary:* Increasing stage associated with poorer survival.

(c) *All sites except testis and reticuloses:* Increasing survival proportions associated with histological gradings—

3→2→1 (Cervix uteri, large bowel, bladder)
3→1→2 (Corpus uteri)
2→3→1 (Ovary)
1→2→3 (Breast)

The differences between Grades 1 and 2 in the corpus uteri site and 2 and 3 in the ovary site are very small.

(d) *Large bowel, bladder, and reticuloses:* Within the bladder site, females have poorer survival. Only marginal differences are present in the remaining relevant sites.

The reanalysis of the breast data confirmed the above conclusions relating to breast site. The coefficients of the dummy variables, however, indicate that patients with no information on either stage, grade or lymphnodes with/without metastases have significantly worse survival.

The further analysis of the cervix uteri data to include duration of symptoms indicated that this factor is not of prog-

TABLE II.—*Estimated model parameters and (in brackets) their standard errors*

| Site | $\alpha$ | DNA coefficients $\beta_1$ | DNA coefficients $\beta_2$ | DNA coefficients $\beta_3$ | Age $\beta_4$ | Stage $\beta_5$ | Grade $\beta_6$ | Grade $\beta_7$ | Sex $\beta_8$ | Lymph nodes $\beta_9$ | Sample size used in estimation |
|---|---|---|---|---|---|---|---|---|---|---|---|
| Cervix uteri | | | | | | | | | | | |
| (a) Squamous-cell carcinoma | 0·78 (0·04) | | | | | | | | | | 431 |
| (b) Adenocarcinoma | 0·71 (0·14) | −0·174 (0·161) | −0·009* (0·004) | −0·005 (0·003) | 1·33** (0·58) | 0·52**** (0·06) | 0·24 (0·22) | 0·45** (0·20) | — | — | 23 |
| (c) Adenoacanthoma | 0·85 (0·11) | | | | | | | | | | 49 |
| Corpus uteri | 0·86 (0·09) | 0·271 (0·771) | 0·037*** (0·014) | 0·033 (0·028) | 12·31**** (2·33) | 0·17 (0·19) | −0·02 (0·36) | 0·82** (0·34) | — | — | 122 |
| Breast | 1·21 (0·12) | 0·188 (0·403) | 0·013 (0·008) | 0·009 (0·008) | 1·69 (1·33) | 0·28* (0·16) | −0·17 (0·42) | −0·82* (0·47) | — | 1·22**** (0·32) | 127 |
| Ovary | 0·89 (0·10) | 0·993 (0·819) | 0·016 (0·009) | 0·007 (0·020) | 3·98** (1·71) | 0·73**** (0·16) | 2·23*** (0·82) | 1·80** (0·83) | — | — | 68 |
| Gastrointestinal tract | | | | | | | | | | | |
| (a) Stomach | 0·07 (0·16) | | | | | | | | | | 11 |
| (b) Colon/caecum | 0·64 (0·09) | 0·171 (0·413) | −0·014 (0·009) | −0·005 (0·009) | 4·55*** (1·45) | — | 0·22 (0·27) | 1·83**** (0·44) | 0·03 (0·23) | — | 57 |
| (c) Rectum | 0·69 (0·09) | | | | | | | | | | 61 |
| Bladder | 0·83 (0·12) | 0·071 (0·609) | −0·003 (0·017) | −0·010 (0·011) | 6·78** (3·43) | — | 0·32 (0·46) | 0·84* (0·51) | −0·51 (0·48) | — | 49 |
| Testis | 0·58 (0·13) | 0·140 (1·604) | 0·015 (0·036) | 0·006 (0·023) | −3·12 (2·34) | — | — | — | — | — | 28 |
| Reticuloses | 0·72 (0·13) | 0·578 (1·068) | −0·006 (0·029) | −0·014 (0·027) | 1·32 (1·01) | — | — | — | −0·02 (0·56) | — | 34 |

Significance ($P$) * < 0·1, ** < 0·05, *** < 0·01, **** < 0·001.

(In the printed table, the DNA coefficient, age, stage, grade, sex and lymph node values for Cervix uteri (a)–(c) and for Gastrointestinal tract (a)–(c) are shared across the bracketed sub-sites.)

TABLE III.—*Estimated model parameters and (in brackets) estimated standard errors in reanalysis of breast data*

| | | | | | | |
|---|---|---|---|---|---|---|
| $\alpha$: | 0·92 | | | | | |
| | (0·07) | | | | | |
| $\beta_1$: | 0·338 | $\beta_2$: | 0·020*** | $\beta_3$: | 0·008 | DNA |
| | (0·319) | | (0·007) | | (0·007) | |
| $\beta_4$: | 1·66 | | | | | age |
| | (1·06) | | | | | |
| $\beta_5$: | 0·32 | | | $\gamma_1$: | 1·58**** | stage |
| | (0·13) | | | | (0·38) | |
| $\beta_6$: | −0·24** | $\beta_7$: | −0·39 | $\gamma_2$: | 1·26*** | grade |
| | (0·35) | | (0·39) | | (0·44) | |
| $\beta_9$: | 1·04**** | | | $\gamma_3$: | 0·79*** | lymph |
| | (0·30) | | | | (0·30) | nodes |

For $j = 1,2,3$, $\gamma_j$ is coefficient of $\gamma_j = \begin{cases} 0 & \text{information available on associated variable} \\ 1 & \text{otherwise} \end{cases}$

Significance ($P$) * < 0·1, ** < 0·05, *** < 0·01, **** < 0·001.

nostic importance, the estimated coefficient of −0·01 having a standard error of 0·01.

### DISCUSSION

Although the complete cytogenetic study of a human tumour requires the detailed and painstaking analysis of its chromosome changes, significant information relating to these changes can readily be obtained from DNA measurements on interphase cells. Moreover, data can easily be obtained with this technique on unselected series of cases, including those in which mitoses are absent from the sample of cells obtained for study. The present results have only established tenuous associations between DNA measurement and prognosis at sites where the tumours can be placed in one or other of 2 "ploidy groups".

Correlations between DNA value and prognosis have been investigated by Tavares *et al.* (1966), who found a relatively poor prognosis for carcinomas of the bladder and prostate when the DNA value was near-triploid or near-hexaploid. The data presented here show that different sites differ both with respect to the form of the distribution curve of DNA values of randomly selected series of tumours and to the relationship between the ploidy group of individual tumours from a given site and their prognosis. *A priori* suggestions that tumours with modal values at or close to the diploid, and perhaps also to the tetraploid level, have a different prognosis from those within that ploidy group but departing substantially from these levels were not supported conclusively. Nevertheless individual trends, some of which approached significance, suggested that tumours close to these euploid levels have a slightly worse prognosis for the cervix uteri, large bowel, bladder and reticuloses, and a slightly better prognosis for the corpus uteri, breast, ovary and testis.

Chromosome analysis using banding techniques is of course a more discriminating as well as a more difficult technique than Feulgen cytophotometry. Whether it will prove more useful as a diagnostic or prognostic guide, for example by enabling a distinction to be made between near-diploid tumours with slight chromosome changes such as one or 2 trisomies or translocations and those with more extensive changes remains to be seen.

We thank Miss K. Lam for drawing the figures, and Mrs B. J. Langdon and Mrs P. Rushmere for secretarial services. This work was supported by a grant from the Cancer Research Campaign.

### REFERENCES

ATKIN, N. B. (1971) Modal DNA value and chromosome number in ovarian neoplasia. A clinical and histopathologic assessment. *Cancer*, **27**, 1064.

ATKIN, N. B. (1972) Modal deoxyribonucleic acid value and survival in carcinoma of the breast. *Br. Med. J.*, i, 271.

ATKIN, N. B. (1973) High chromosome numbers of seminomata and malignant teratomata of the testis: A review of data on 103 tumours. *Br. J. Cancer*, **28**, 275.

ATKIN, N. B. (1976*a*) Prognostic significance of ploidy level in human tumours I: Carcinoma of the uterus. *J. Natl Cancer Inst.*, **56**, 909.

ATKIN, N. B. (1976*b*) Prognostic significance of ploidy level in human tumours II: Extra-uterine cancers and summary of data on 1171 tumours. *Cytobios*, **15**, 233.

ATKIN, N. B. & RICHARDS, B. M. (1956) Deoxyribonucleic acid in human tumours as measured by microspectrophotometry of Feulgen stain: A comparison of tumours arising at different sites. *Br. J. Cancer*, **10**, 769.

ATKIN, N. B. & RICHARDS, B. M. (1962) Clinical significance of ploidy in carcinoma of cervix: Its relation to prognosis. *Br. Med. J.*, ii, 1445.

ATKIN, N. B., MATTINSON, G. & BAKER, M. C. (1966) A comparison of the DNA content and chromosome number of fifty human tumours. *Br. J. Cancer*, **20**, 87.

COX, D. R. (1972) Regression models and life tables. *J. R. Statist. Soc.*, B, **34**, 187.

KAPLAN, E. L. & MEIER, P. (1958) Non-parametric estimation from incomplete observations. *J. Am. Statist. Assoc.*, **53**, 457.

KAY, R. (1977) Proportional hazard regression models and the analysis of censored survival data. *Appl. Statist.*, **26**, 227.

PRENTICE, R. L. (1973) Exponential survivals with censoring and explanatory variables. *Biometrika*, **60**, 279.

TAVARES, A. S., COSTA, J., DE CARVALHO, A. & REIS, M. (1966) Tumour ploidy and prognosis in carcinomas of the bladder and prostate. *Br. J. Cancer*, **20**, 438.

WILLIAMS, J. S. (1978) Efficient analysis of Weibull survival data from experiments on heterogeneous patient populations. *Biometrics*, **34**, 209.